# Upper gastrointestinal Crohn's disease: What are we talking about?

**DOI:** 10.1002/ueg2.12664

**Published:** 2024-11-07

**Authors:** Luisa Bertin, Brigida Barberio, Edoardo Vincenzo Savarino

**Affiliations:** ^1^ Gastroenterology Unit Azienda Ospedale Università Padova Padova Italy; ^2^ Department of Surgery, Oncology and Gastroenterology University of Padova Padova Italy

**Keywords:** definition, diagnosis, esophagus, gastric Crohn's disease

Crohn's disease (CD) is a chronic inflammatory condition that can affect any part of the gastrointestinal (GI) tract, from the mouth to the anus.^1^ While lower GI involvement is more commonly recognized, upper GI (UGI) CD is gaining increasing attention despite its relatively uncommon presentation in adults.

The prevalence of UGI involvement in CD varies widely, with reported rates ranging from 0.05% to 83%, due to differing definitions and diagnostic criteria across studies.^2^ In fact, some studies report much higher rates when upper endoscopy is routinely performed, highlighting the potential underdiagnosis of this condition. Of note, a recent study found that while 41% of newly diagnosed adult CD patients had UGI involvement, only 32% had corresponding symptoms, raising questions about the utility of additional investigations, and thereafter treatment, when lesions are asymptomatic.^3^ The lack of specific symptomatology likely contributes to the under‐recognition of UGI CD, leading to either a delayed diagnosis until the disease progresses to the lower GI tract or the misattribution of symptoms to lower GI disease.

The definition of UGI CD is complex and varies across studies. In this issue of the *United European Gastroenterology Journal,* a scoping review by Yuan and colleagues attempts to address these inconsistencies by analyzing key guidelines and consensus papers in order to stimulate future research in this area.^4^ The review highlights that while the Montreal classification primarily categorizes UGI CD as L4, affecting segments up to the proximal ileum, organizations like the European Crohn's and Colitis Organisation and the European Society of Gastrointestinal and Abdominal Radiology include the esophagus, stomach, and duodenum in their definitions of UGI CD.^5^, ^6^ Notably, the review also highlights the absence of a clear definition in pediatric guidelines, which is particularly concerning given that significant small intestine involvement in pediatric CD patients can lead to serious consequences such as growth failure and stricturing disease. Moreover, the importance of including biopsies from the UGI tract in clinical trials involving children is underscored, as this could lead to a more accurate assessment. In adults, on the other hand, the role of histological findings in the absence of corresponding endoscopic or clinical evidence remains uncertain, complicating diagnostic and management strategies.

The precise definition of UGI CD is critical, as it influences diagnostic approaches, prevalence estimates, prognostic implications, and treatment responses. Regarding prognosis, CD involving the jejunum and proximal ileum often presents later in the disease course and is associated with a stricturing phenotype that frequently necessitates surgical intervention. This involvement has been identified as an independent risk factor for surgery, hospitalization, and overall complications, in contrast to disease located in the upper GI tract.^2^ As to the diagnosis, CD assessment in the UGI tract still remains a significant task, particularly due to the limited accessibility of segments like the jejunum with standard endoscopic techniques. There is growing recognition that diagnosing CD beyond the duodenum may require more advanced diagnostic approaches, including capsule endoscopy, device‐assisted enteroscopy, or MRI. Moreover, the endoscopic appearance of UGI lesions in CD is highly variable, ranging from aphthae, erosions, and ulcerations to bamboo joint‐like lesions, strictures, and notch‐like formations.^7^ This variability has sparked debate about which is the best approach to diagnose UGI CD: whether UGI CD should be confirmed histologically, through endoscopic and radiologic findings alone, or if histology alone might suffice. Additionally, while there is a scoring system for capsule endoscopy in CD, no such scoring system exists for gastroscopy, further complicating a standardized diagnostic approach and management.

Finally, the uncommon occurrence of UGI involvement in CD has resulted in a scarcity of data concerning its pharmacological treatment. Clinical trials specifically evaluating the effectiveness of biologic therapies for UGI CD are scant, and evidence is often limited to case reports or case series.^8^ Treatment strategies often focus on controlling coexisting distal disease.^9^ Surgical intervention for UGI CD, particularly in cases of stricturing disease, presents additional challenges, with techniques such as Roux‐en‐Y gastrojejunostomy being utilized in some instances. However, the choice of this intervention is based on limited evidence and is not supported by established guidelines.^10^

In summary, the diagnosis and management of UGI CD are difficult due to limited evidence and the heterogeneous nature of the disease. Future studies should focus on standardizing definitions, understanding the impact of histological activity, and developing more effective treatment strategies for patients with UGI involvement. Standardized diagnostic criteria and targeted therapeutic strategies will be crucial at improving outcomes for this under‐recognized subset of CD patients.

## AUTHOR CONTRIBUTIONS

Luisa Bertin, Brigida Barberio and Edoardo Vincenzo Savarino: collection and analysis of the data, draft of the manuscript, approving final version.

## Data Availability

Data sharing is not applicable to this article as no new data were created or analyzed in this study.

## References

[1] Roda G , Chien NS , Kotze PG , et al. Crohn’s disease. Nat Rev Dis Primers. 2020;6:1–19. 32242028 10.1038/s41572-020-0156-2

[2] Chin YH , Ng CH , Lin SY , Jain SR , Kong G , Koh JWH , et al. Systematic review with meta‐analysis: the prevalence, risk factors and outcomes of upper gastrointestinal tract Crohn’s disease. Dig Liver Dis. 2021;53(12):1548–1558. 10.1016/j.dld.2021.07.037 34412995

[3] Horjus THCS , Meijer J , Rovers L , et al. Prevalence of upper gastrointestinal lesions at primary diagnosis in adults with inflammatory bowel disease. Inflamm Bowel Dis. 2016;22:1896–1901. 27057685 10.1097/MIB.0000000000000786

[4] Yuan Y , Sedano R , Solitano V , Nardone OM , Crowley E , Jairath V . Scoping review: navigating the ‘upper’ in upper gastrointestinal crohn's disease. United European Gastroenterol J. In press. 10.1002/ueg2.12697 PMC11652330 39541219

[5] Satsangi J , Silverberg MS , Vermeire S , et al. The Montreal classification of inflammatory bowel disease: controversies, consensus, and implications. Gut. 2006;55(6):749–753. 10.1136/gut.2005.082909 16698746 PMC1856208

[6] Maaser C , Sturm A , Vavricka SR , Kucharzik T , Fiorino G , Annese V , et al. ECCO‐ESGAR Guideline for Diagnostic Assessment in IBD Part 1: initial diagnosis, monitoring of known IBD, detection of complications. J Crohns Colitis. 2019;13(2):144–164K. 10.1093/ecco-jcc/jjy113 30137275

[7] Graca‐Pakulska K , Błogowski W , Zawada I , Deskur A , Dąbkowski K , Urasińska E , et al. Endoscopic findings in the upper gastrointestinal tract in patients with Crohn’s disease are common, highly specific, and associated with chronic gastritis. Sci Rep. 2023;13(1):703. 10.1038/s41598-022-21630-5 36639398 PMC9839771

[8] Vale Rodrigues R , Sladek M , Katsanos K , van der Woude CJ , Wei J , Vavricka SR , et al. Diagnosis and outcome of oesophageal Crohn’s disease. J Crohns Colitis. 2020;14(5):624–629. 10.1093/ecco-jcc/jjz201 31837220

[9] Orrell M , van ’t Hullenaar C , Gosling J . Upper gastrointestinal tract involvement in Crohn’s disease: a case report. Int J Surg Case Rep. 2021;81:105810. 33887830 10.1016/j.ijscr.2021.105810 PMC8041720

[10] Adamina M , Bonovas S , Raine T , Spinelli A , Warusavitarne J , Armuzzi A , et al. ECCO guidelines on therapeutics in Crohn’s disease: surgical treatment. J Crohns Colitis. 2020;14(2):155–168. 10.1093/ecco-jcc/jjz187 31742338

